# Are there educational disparities in health and functioning among the oldest old? Evidence from the Nordic countries

**DOI:** 10.1007/s10433-019-00517-x

**Published:** 2019-06-17

**Authors:** Linda Enroth, Marijke Veenstra, Marja Aartsen, Agnete Aslaug Kjær, Charlotte Juul Nilsson, Stefan Fors

**Affiliations:** 1 Faculty of Social Sciences (Health Sciences) and Gerontology Research Center (GEREC), Tampere University, Tampere, Finland; 2 Norwegian Social Research, OsloMet - Oslo Metropolitan University, Oslo, Norway; 3 The Danish Center for Social Science Research (VIVE), Department of Political Science, University of Copenhagen, Copenhagen, Denmark; 4 Section of Social Medicine, Department of Public Health, University of Copenhagen, Copenhagen, Denmark; 5 Aging Research Center, Karolinska Institutet and Stockholm University, Stockholm, Sweden

**Keywords:** Inequality, Self-rated health, Functioning, Oldest old, Nordic countries, Prospective meta-analysis

## Abstract

With the ageing of the population and recent pressures on important welfare state arrangements, updated knowledge on the linkage between socioeconomic status and health in old age is pertinent for shedding light on emerging patterns of health inequalities in the Nordic countries. This study examined self-rated health (SRH), mobility and activities of daily living (ADL) according to level of education in the three oldest old age groups 75–84, 85–94, and 95+, in four Nordic countries. Altogether, 6132 individuals from Danish Longitudinal Study of Ageing, Norwegian Life Course, Ageing and Generation study, Swedish Panel Study of Living Conditions of the Oldest Old, the 5-Country Oldest Old (Sweden) and Vitality 90 + Study were analysed. First, associations of education level with SRH, mobility, and ADL were estimated for each individual study by means of age- and gender-adjusted logistic regression. Second, results from individual studies were synthesized in a meta-analysis. Older adults with higher education level were more likely to report good SRH, and they were more often independent in mobility and ADL than those with basic education when all age groups were combined. In mobility and ADL, differences between education groups remained stable across the age groups but for SRH, differences seemed to be weaker in older ages. With only a few exceptions, in all age groups, individuals with higher education had more favourable health and functioning than those with basic education. This study shows remarkable persistence of health and functioning inequalities in the Nordic countries throughout later life.

## Introduction

Socioeconomic health inequality, i.e. higher life expectancy and better health in higher socioeconomic status (SES) groups, is a global phenomenon and the Nordic countries (Denmark, Finland, Iceland, Norway and Sweden), known for their generous welfare systems, are no exceptions (Mackenbach 2012). While the level of generosity, spending on eldercare and the way of organizing health and social services differ in many respects, the Nordic countries have certain social policies in common, such as universal health- and social care coverage and minimum pensions that support longevity and social and economic security in old age (Lundberg et al. 2008; Szebehely and Meagher 2017). Even with this social protection and class-equalizing potential, several studies have shown substantial socioeconomic health inequalities in young, working age and young old age populations in the Nordic welfare states (Bambra 2012; Lahelma et al. 2001; Mackenbach et al. 2008). Yet, relatively little is known about the extent of health inequalities in very old age.

Besides the similarities in welfare state models, Nordic countries have rather similar patterns of population ageing. The proportion of those who reach old age (e.g. 75+ or 95+) is smaller in Finland, especially among men, than in the other Nordic countries. However, after reaching old age (i.e. 75 years) the remaining life expectancies are very similar (Jørgensen et al. 2018) ranging from 12.04 to 12.67 years (Human Mortality Database 2018). Altogether, the proportion of 75 + year olds of the total population is expected to increase from 7.5% in 2015 to 12% in 2040 in the Nordic countries (Eurostat 2018).

House et al. (1990) have elaborated on the potential mechanisms by which the association of SES with health may vary by age. In young age groups, the prevalence of health problems is lower due to biological robustness, which may lead to smaller health inequalities. In middle and young old age, the impact of work-related psychosocial and environmental risk factors on health is high, causing higher inequality in health between socioeconomic groups. Different hypotheses have been put forward on the mechanisms of the increase or decrease in health inequalities in old age, see e.g. (Hoffmann 2011; Rehnberg et al. 2019). Exit from the labour market reduces exposure to work-related, unequally distributed health risks, and this may lead to a convergence in health inequalities in later life (House et al. 1994). In addition, inevitable biological processes and the equalizing social policies in welfare states are suggested to contribute to the decline in health inequalities in old age. Counteracting with the biological processes, also selective survival, which refers to the higher mortality in lower socioeconomic groups, may result in reduced health inequalities if only the healthiest and most robust individuals from the lower socioeconomic groups reach old age (Dupre 2007). On the other hand, if the accumulation of advantage or disadvantage in material and social resources lasts throughout life, it could lead to an increase in health inequalities in old age (Ross and Wu 1996). Further, if the health effects of poor working conditions are postponed to older ages, it might increase health inequalities in old age.

The evidence of the existence and size of health inequalities by age in later life is inconsistent, and has barely touched upon the oldest old age groups. Minkler et al. (2006) showed a social gradient in functional limitations among 55–84 year olds in the US: the lower the level of education, the worse the functioning. Inequalities were strongest in younger age groups (55–64) and flattened towards older age groups, and finally disappeared at the age of 85+. Huisman et al. (2003) also showed decreasing but persistent inequalities in long-term disabilities and self-rated health (SRH) for men in the age groups 60–69, 70–79, and 80+, but not for women in the oldest age group in a study with 11 European countries. Rostad et al. (2009) who studied inequalities in limiting long standing illnesses and SRH for women in Norway found that inequalities were apparent and even seemed to increase in SRH in the oldest age group (85+) according to education, but not with other health or SES indicators. Arber and Cooper (1999) found persistent inequalities in disability (80+) and in SRH (85+) in Britain with previous occupation as a SES indicator. Schöllgen et al. (2010) studied health inequalities in physical, functional and subjective health with education, income and financial assets as SES indicators in the study sample that consisted of 40–85 year-old Germans. The study showed in general stability or increase in inequalities throughout the age groups (40–54, 55–69, and 70–85) but decrease according to education in subjective health. Other earlier studies that have shown health inequalities among the oldest old have been limited to one cohort or one country (Bootsma-van Der Wiel et al. 2005; Enroth et al. 2013; Fors and Thorslund 2015).

With the ageing of the populations and recent pressures on important welfare state arrangements, updated knowledge on the linkage between SES and health in old age is pertinent for shedding light on emerging patterns of health inequalities in the Nordic countries. The current study focuses on health and functioning inequalities by the level of education. We assess the extent of health and functioning (self-rated health, independence in mobility and activities of daily living (ADL)) inequalities among people aged 75+ in four Nordic countries (Denmark, Finland, Norway and Sweden). The study aims to provide an overview of the direction and magnitude of health and functioning inequalities in three age groups 75–84, 85–94, and 95+, and in the 75+ population as a whole.

## Methods

### Study samples

This study utilized data from the Danish Longitudinal Study of Ageing (DLSA) from Denmark, the Vitality 90+ Study from Finland, the Norwegian Life Course, Ageing and Generation study (NorLAG) from Norway, the Swedish Panel Study of Living Conditions of the Oldest Old (SWEOLD), and the Swedish part of the 5-Country Oldest Old Project (SE-COOP) from Sweden. All studies have approval from the local ethical committees, and an informed consent was obtained from all study participants.

*DLSA* includes a representative sample of the Danish population aged 52 and above from nine selected cohorts. It is a longitudinal study with five waves. For this study, we included participants from birth cohorts 1920, 1925, 1930 and 1935 who responded to the fourth wave of data collection in 2012/2013. The response rate for 1780 individuals aged 77, 82, 87 and 92 was 73.7%. The response rate for the total population in the fourth wave was 74.7% (Kjær et al. 2019).

*Vitality 90*+ *Study* is based on the 90 + population in the third largest city in Finland. The number of participants was 1277, with a response rate of 79.5% in 2010, and 1637 with a response rate of 80% in 2014 (Jylhä et al. 2013). Data from the 2014 wave were analysed only for the 85–94 age group because there was an extensive overlap of participants with 2010 data for the 95+ age group.

*NorLAG* includes a random stratified sample of the Norwegian population aged 45–85. The response rate for the whole sample in 2007–2008 was 71.6%. For the purposes of the present study, we included 850 participants aged 75–84 with a response rate of 57.9% (Bjørshol et al. 2007; Slagsvold et al. 2012).

*SWEOLD 2014* is based on a random sample of the Swedish population aged 69 and over. The response rate was 84.3%. The number of participants (75 years and over) included in the current study was 868. Since 85+ year olds were oversampled in the data collection, weights were used in the analyses to account for the differences in sampling probability (Lennartsson et al. 2014).

*SE*-*COOP* is based on a random sample of 320 centenarians (interviewed during their 100^th^ year) in Sweden in 2011–2012, with a response rate of 85.9% (*n* = 274). Since men were oversampled in the data collection, weights were used in the analyses to account for differences in sampling probability (Parker et al. 2014).

In total, the study population from five studies comprised 6132 individuals of which 2111 were men and 4021 women (Table 1). The total population was stratified into three age groups where the number of participants was 2760 (women 55%) in the age group of 75–84 years old, 2789 (women 74%) in the age group of 85–95 years old and 583 (women 77%) in the age group of 95+ years old.

**Table 1:** Description of the study samples

Name of the study	DLSA	NorLAG	SWEOLD	SE-COOP	Vitality 90+	
Country	Denmark	Norway	Sweden	Sweden	Finland	Finland
Year of the study	2012/2013	2007/2008	2014	2011/2012	2010	2014
Response rate %	73.7^a^	57.9^a^	84.3^b^	85.9^c^	79.5^c^	80.0^b^
Age group 75–84, *n*	1372	844	544			
Men, *n* (%)	608 (44.3)	395 (46.8)	247 (45.4)			
Women, *n* (%)	764 (55.7)	449 (53.2)	297 (54.6)			
Age, median, min–max	77, 77–82	78, 75–84	79, 75–84			
Basic education, *n* (%)	655 (47.7)	280 (33.2)	257 (47.2)			
Higher education, *n* (%)	717 (52.3)	564 (66.8)	287 (52.8)			
Proxy respondent, *n* (%)			42 (7.7)			
Age group 85–94, *n*	408		244		971	1166
Men, *n* (%)	141 (34.6)		95 (38.9)		194 (20)	297 (25.5)
Women, *n* (%)	267 (65.4)		149 (61.1)		777 (80)	869 (74.5)
Age, median, min–max	87, 87–92		88, 85–94		91, 90–94	91, 90–94
Basic education, *n* (%)	224 (54.9)		142 (58.2)		523 (53.9)	585 (50.2)
Higher education, *n* (%)	184 (45.1)		102 (41.8)		448 (46.1)	581 (49.8)
Proxy respondent, *n* (%)			57 (23.4)		134 (13.9)	188 (16.3)
Age group 95+, *n*			53	272	258	
Men, *n* (%)			21 (39.6)	75 (27.6)	38 (14.7)	
Women, *n* (%)			32 (60.4)	197 (72.4)	220 (85.3)	
Age, median, min–max			96, 95–105	100	96, 95–107	
Basic education, *n* (%)			39 (73.6)	199 (73.2)	171 (66.3)	
Higher education, *n* (%)			14 (26.4)	73 (26.8)	87 (33.7)	
Proxy respondent, *n* (%)			17 (32.1)	110 (40.2)	69 (26.8)	

^a^Response rate only for participants included in this study, ^b^Response rate for the total population in the study (SWEOLD excluded from this study younger than 75 years old and The Vitality 90+ Study 2014 individuals 95 years and older), ^c^Response rate for the participants in this study is the same as for the total population

The main mode of data collection was face-to-face interviews in SWEOLD and SE-COOP. However, the studies were complemented with telephone interviews when preferred by the respondents. For the Vitality 90+ Study, the data were collected using mailed surveys, and for the NorLAG computer-assisted telephone interviews (CATI) were carried out followed by a mailed survey. For DLSA, data were mainly collected with phone interviews, but the respondents were offered a chance for a personal home visit if they were not capable of answering by phone. In addition, it was possible to use proxy respondents in SWEOLD, SE-COOP and in the Vitality 90+ Study. In all five studies, both community dwellings and institutionalised individuals were included. However, institutionalised were generally under-represented in the DLSA and NorLAG.

### Measures

Social stratification was measured with the highest attained level of education. Education is a commonly used measure also among older people (Huisman et al. 2003; Schöllgen et al. 2010), and it is highly comparable across the Nordic countries. Since the level of education is in general low among the oldest old, we dichotomized it distinguishing between basic education and more than basic education. Basic education refers to primary education, which for the birth cohorts in this study varied between 6 and 7 years in the Nordic countries. Higher education refers to more than 7 years of education or to vocational or at least secondary education. Information on the level of education came from the surveys, except for DLSA, where information on highest attained educational level was gathered from the national registers in combination with information from the survey interviews.

We assessed health and functioning with three measures that are commonly used in surveys directed to older population self-rated health (SRH), mobility and activities of daily living (ADL). SRH is a general measure of health status for which individuals take into account e.g. subjective feelings such as pain, diagnoses and medication, problems in functioning, health behaviour and age (Jylhä 2009). SRH is a well-established predictor of mortality and is shown to be associated e.g. with the number of diagnoses and medications among the oldest old (Bravell et al. 2011). SRH was assessed with the question: How would you assess your general state of health? The answer options were: (1) really good, (2) good, (3) fair, (4) poor, or (5) very poor for DLSA; (1) excellent, (2) very good, (3) good, (4) fair, or (5) poor for NorLAG; (1) excellent, (2) good, (3) acceptable, (4) poor or (5) very poor for SE-COOP; (1) good, (2) neither good nor bad, or (3) bad for SWEOLD; and (1) good, (2) fairly good, (3) average, (4) fairly poor or (5) poor for Vitality 90+ Study. Good SRH was indicated with answer options 1 and 2 in DLSA, SE-COOP and Vitality 90+ Study; 1–3 in NorLAG; and 1 in SWEOLD. Proxy respondents were excluded from the analyses of SRH because of the subjective nature of the question.

In the literature, ADL and mobility summary measures often vary in the number of items. However, they reflect coping at home and need of help, and are shown to be associated with the performance tests among the oldest old (Bravell et al. 2011). The psychometric properties for ADL indicators, such as reliability and validity, have been shown to be reasonably good for the 5-item Katz list (Hopman-Rock et al. 2018), and Rodgers and Miller (1997) showed that a composite measure of ADL had high predictive validity also with a smaller subset of ADL indicators. ADL was assessed by the self-reported ability to get in and out of bed, and to dress and undress in NorLAG, Vitality 90+ Study, SWEOLD, and in SE-COOP; while in DLSA, the question on the ability to get in and out of bed was substituted with a question on the ability to wash or shower. Those able to perform both activities without help of another person were considered as being independent in ADL. Mobility was assessed by the self-reported ability to perform two or three activities, depending on the study. DLSA, Vitality 90+ Study and SWEOLD included questions on the ability to walk indoors, walk 400/500 metres or walk around outdoors, and use stairs. NorLAG and SE-COOP included questions on the ability to walk 400/500 metres and use stairs. Those able to perform all (two or three) activities without difficulty or limitation (NorLAG), without help of another person (DLSA, Vitality 90+ Study, SWEOLD), and with or without difficulty (SE-COOP), were considered as being independent in mobility.

As earlier literature shows (Enroth et al. 2013), age and gender are potential confounders in the association between education level and health and functioning among the oldest old. According to that, age and gender were included in the analyses as covariates.

### Statistical analyses

The first step of the analyses was to assess the unadjusted prevalences of good SRH and independence in mobility, and ADL by level of education. Second, we used logistic regression analysis to assess whether SRH and functioning differed between levels of education. Independent variable (level of education) was entered simultaneously with the covariates (age and gender) into the regression model. The estimates are presented as odds ratios (OR) with 95% confidence intervals (CI). The analyses were conducted separately for three age groups 75–84 (DLSA, NorLAG, and SWEOLD), 85–94 (DLSA, Vitality 90+ Study 2010 and 2014, and SWEOLD), and 95+ years old (Vitality 90+ Study 2010, SWEOLD, and SE-COOP). The country-specific analyses, based on individual data, were run separately in each country. Finally, a meta-analysis (MA) was used for synthesizing the results from regression analyses. Statistical significance was set to *p* < 0.05 in all analyses and the analyses were conducted with the statistical software Stata version 14.

Often, a MA is conducted to summarize existing literature with the aim to aggregate findings (Ghersi et al. 2008). Since existing research is limited and the comparability of the studies is weak, we provided the input for the MA ourselves (prospective meta-analysis). It gave us the possibility to pre-plan the method of analysis, definitions of SES and health as well as the included studies. MA synthesize results across the studies and gives the direction and effect size of the findings on a common scale. Because the sampling frame included multiple populations from Nordic countries, random effects models were used. The model assumes that true effect size varies from study to study and the summary effect, which the analysis provides, is an estimate of the mean of a distribution of true effects (Borenstein et al. 2010). Consistency of the effects across the samples, i.e. between-study heterogeneity, was tested using the Q statistic, and quantified by the I-squared value. I-squared describes the percentage of total variation across studies that is due to heterogeneity rather than chance. The crude categories for heterogeneity have been defined as low with an I-squared value of 0–25%, moderate with an I-squared of 25–75%, and high with an I-squared of 75–100%. (Higgins et al. 2003).

## Results

The unadjusted prevalences showed that, in general, individuals in the youngest age group had a higher percentage of being independent in mobility and in ADL than individuals in the middle age group. In turn, the percentage was higher in the middle age group than in the oldest age group (Table 2). The DLSA study tended to show a higher level of independence in mobility than the other studies. For SRH, the variability between age groups and between countries was high, especially in the oldest age group. Participants in the Vitality 90+ Study assessed their health worse than participants in the other studies. Overall, with only a few exceptions, in all age groups, for all three outcomes, and for all countries, individuals with higher education had more favourable health and functioning than those with basic education.

**Table 2:** Prevalence of good self-rated health (SRH), independence in mobility and activities of daily living (ADL) by education for age groups 75–84, 85–94 and 95+

Education	75–84 years old							
	DLSA		NorLAG		SWEOLD			
	Basic	Higher	Basic	Higher	Basic	Higher		
SRH %								
Good	57.5	70.5	51.6	62.1	37.9	60.7		
Mobility %								
Independent	90.2	93.4	52.0	58.7	47.0	60.1		
ADL %								
Independent	93.4	95.9	96.7	97.9	89.4	94.7		

Education	85–94 years old							
	DLSA		Vitality 90+ (2010)		Vitality 90+ (2014)		SWEOLD	
	Basic	Higher	Basic	Higher	Basic	Higher	Basic	Higher
SRH %								
Good	58.6	53.9	23.4	31.8	23.9	35.4	42.4	48.4
Mobility %								
Independent	78.8	78.1	39.6	52.0	44.5	53.9	20.9	47.2
ADL %								
Independent	81.9	82.6	77.7	83.3	76.3	80.8	67.6	82.8

Education	95+ years old							
	Vitality 90+ (2010)		SWEOLD		SE-COOP			
	Basic	Higher	Basic	Higher	Basic	Higher		
SRH %								
Good	15.5	18.8	45.0	60.5	79.3	80.4		
Mobility %								
Independent	17.1	33.3	10.5	22.7	31.2	44.2		
ADL %								
Independent	58.0	73.3	62.1	70.2	50.4	66.7		

**Fig. 1:**
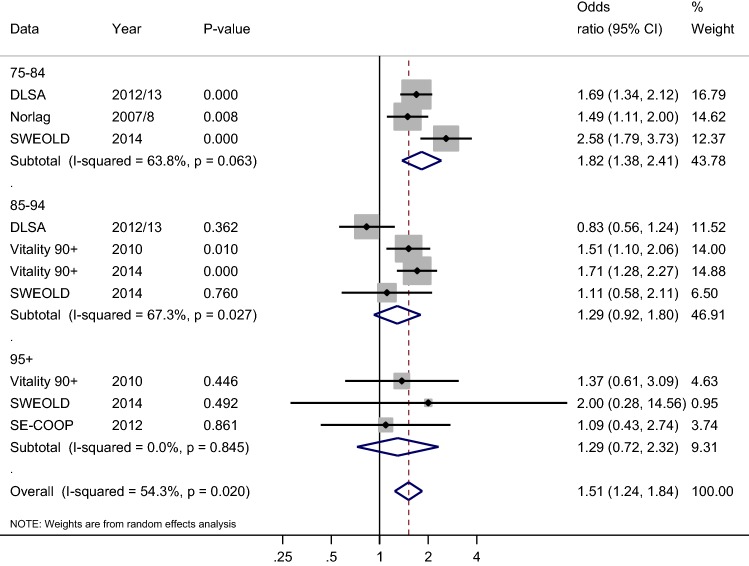
Forest plot on good self-rated health (SRH) according to education (ref. basic education). Odds ratios are adjusted for age and gender. Pooled odds ratios (diamonds) are presented separately for 75–84, 85–94, and 95+ age groups and for all studies combined

Figure 1 shows a forest plot for good SRH according to level of education separately for all age groups and an overall summary effect for all age groups combined. The age-specific analysis for the youngest age group (75–84 years old) showed significantly higher odds for good SRH among those with higher education than among those with basic education in all individual studies and in the summary effect that combines all studies (OR 1.82 CI 1.38; 2.41). For the middle age group (85–94 years old), the DLSA study differed from the other studies by showing lower odds for good SRH for those with higher education; however, this result was not statistically significant. For the oldest age group (95+ years old) all individual studies showed higher odds for good SRH for those with higher education. However, the summary effect did not reach statistical significance neither for the middle age group nor for the oldest age group. When synthesizing results for all age groups, people with higher education had higher odds of having good SRH than those with basic education (OR 1.51 CI 1.24; 1.84). The overall between-study heterogeneity showed moderate consistency for the studies (*p* = 0.02, I-squared 54.3%).

**Fig. 2:**
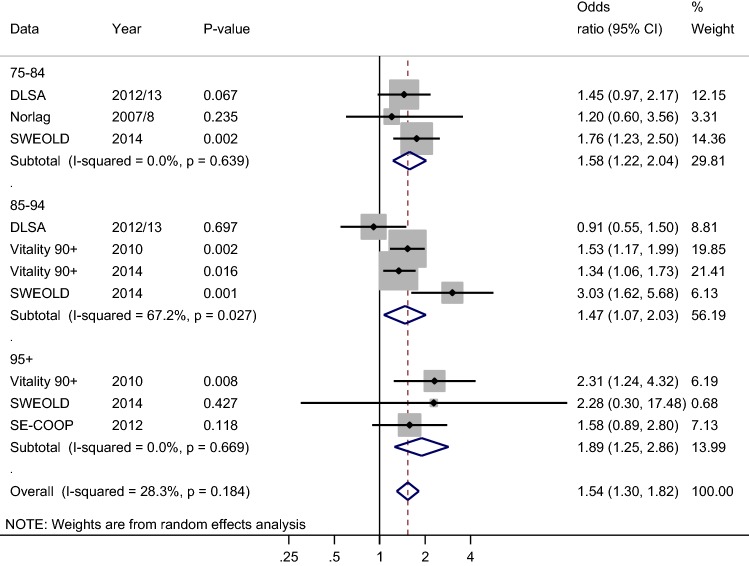
Forest plot on being independent in mobility according to education (ref. basic education). Odds ratios are adjusted for age and gender. Pooled odds ratios (diamonds) are presented separately for 75–84, 85–94, and 95+ age groups and for all studies combined

Figure 2 shows a forest plot for mobility. For the youngest age group, all individual studies showed higher independence in mobility for those with higher education when compared to those with basic education. Even though the result was statistically significant only for the SWEOLD study, the summary effect of all studies reached statistical significance (OR 1.58 CI 1.22; 2.04). For the middle age group, all studies except the DLSA showed significantly higher independence in mobility for those with higher education. Moreover, the summary effect showed significant differences in mobility between individuals with higher and basic education (OR 1.47 CI 1.07; 2.03). The results were highly similar for the oldest age group (OR 1.89 CI 1.25; 2.86). Furthermore, synthesized results for all age groups showed the same; higher independence in mobility for those with higher education (OR 1.54 CI 1.30; 1.82). The heterogeneity of all studies was moderate (*p* = 0.18, I-squared 28.3%).

**Fig. 3:**
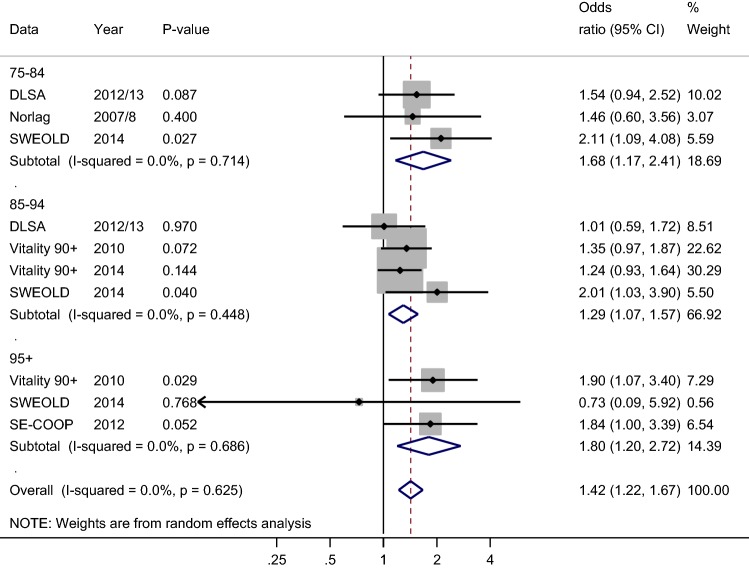
Forest plot on being independent in ADL according to education (ref. basic education). Odds ratios are adjusted for age and gender. Pooled odds ratios (diamonds) are presented separately for 75–84, 85–94, and 95+ age groups and for all studies combined

Figure 3 shows a forest plot for independence in ADL. In the youngest age group, individuals with higher education had higher odds of being independent in ADL than individuals with basic education. The differences in ADL between levels of education were statistically significant in the SWEOLD study, and in the summary analysis (OR 1.68 CI 1.17; 2.41) that included results of all studies in the age group. Also for the middle age group, all individual studies showed higher independency in ADL for those with education beyond the basic level resulting in a statistically significant summary effect (OR 1.29 CI 1.07; 1.57). For the oldest age group, Vitality 90+ Study and SE-COOP studies showed higher odds for being independent in ADL for those with higher education. The result from the SWEOLD study differed from the other studies; however, it had less weight in the analysis because of the small study sample. Thus, the summary effect showed higher independence in ADL for those with higher education also in the oldest age group (OR 1.80 CI 1.20; 2.72). When all studies in all age groups were combined, those with higher education had statistically significantly higher odds (OR 1.42 CI 1.22; 1.67) of being independent in ADL than those with basic education. The between-study variability was low (*p* = 0.63, I-squared 0%).

## Discussion

This study adds knowledge of health and functioning inequalities in very old age by examining inequality patterns according to level of education in three age groups 75–84, 85–94, and 95+ in four Nordic countries. We found that individuals with higher education were more likely to report good SRH, and they were more often independent in mobility and ADL than people with basic education. Stratified analyses across three age groups showed similar findings, except for SRH, where the summary effect was statistically significant only for the age group 75–84. As the study was based on the prospective meta-analysis, it included high-quality comparable data of the oldest old.

We used the same indicators of health and functioning for all age groups across the five studies. When looking at the results of being independent in ADL and mobility, the summary effects show significantly higher independence for people with higher education across all three age groups. For SRH, these differences between education groups were only significant for the youngest age group. SRH is a widely used measure of general health status (Idler and Benyamini 1997), but its interpretation is more complex than for ADL and mobility. The SRH measure is affected by age and culture but also the way of posing the question and answer alternatives (Jylhä 2009). It has been suggested that the oldest old give more positive health ratings compared to younger age groups, which could lead to reduced inequalities. The more positive health ratings may be related to selective mortality, to lowered health expectations in very old age or to downward comparisons, i.e. comparing health to age peers with health problems (Cheng et al. 2007; Tornstam 1975). Another explanation for the slightly deviating patterns of inequality in SRH is that responses to SRH do not include proxy respondents. Smaller sample sizes or greater attrition among those with poor health and basic education would make it more difficult to identify inequalities in SRH in the oldest age groups.

In the individual Nordic studies, the prevalences of being independent in ADL and mobility were very high in the youngest age group especially in NorLAG and DLSA. Both studies showed higher independence for people with higher education but results did not reach statistical significance. The low prevalence of ADL problems in these two studies is related to the strong reliance on community based sampling, which implies a weaker representativeness of individuals not living independently. Kelfve (2017) has elaborated on the representativeness of study populations among older adults, and its effect on the observed magnitude of health inequalities. Her study showed that excluding institutionalised individuals, or those for whom someone else answered the questions (proxy respondent) led to underestimations of the health inequalities.

Our study showed stable inequalities in ADL and mobility until the oldest age group (95+), which is in line with Arber and Cooper (1999) and Schöllgen et al. (2010) but differs from Minkler et al. (2006). This comparison is not optimal since the previous studies had a focus on younger age groups. They also excluded institutionalised, which may explain the difference between this study and Minkler et al. (2006). In SRH analyses, between-study variability was higher, and unlike Rostad et al. (2009), we found slight decrease in inequalities towards higher age groups. One difference between studies was that we categorized education in two groups and the other study used three categories where the highest education group clearly differed from the lowest group. In studying very old people, who generally have a low level of education, the use of a dichotomized measure of education may be justified, although it may ignore some important variation in the social environment.

All five studies had good or excellent response rates. It is, however, known that the study non-respondents tend to have worse health than the study respondents and that poor health is associated with a lower level of education. Thus, it is likely that we underestimate inequalities and as such provide a lower bound for the extent of health inequalities in old age. Moreover, since people with higher education, on average, have longer lives (Moe et al. 2012), their proportion in the study sample is relatively higher than for people with basic education. Selective mortality eliminates frail and unhealthy individuals from a cohort, making initially disadvantaged groups appear compositionally advantaged over time and, thereby, attenuating or even reversing the initial associations (Ferraro and Shippee 2009). If this process occurs at a faster rate among those with lower education, compared to those with more education, this could lead to decreasing health inequalities in old age.

The advantages of this study were the use of synthesized data from four Nordic countries including high number of oldest old and the use of similar variables and methods of analyses. There were also limitations in the study. The study design was cross-sectional and descriptive in the sense that we were not accounting for several possible confounders such as intelligence or personality, that can affect both the level of education and health. Therefore, it is not possible to draw any conclusions about the causal nature of the associations based on our results. Our results show health inequalities across age groups at one time point. Results may reflect continuity of health inequalities with ageing but it can also reflect a cohort effect. In addition, as a methodological consideration, we assessed between-study heterogeneity with Q statistics and I-squared test, which both include uncertainty in a meta-analysis with a low number of studies. High between-study heterogeneity questions whether the summary estimate shows the right magnitude of inequalities since the consistency of the studies is weaker (Borenstein et al. 2010). The heterogeneity statistics from the meta-analysis coincide well with the known differences between the studies. Heterogeneity in SRH, when all age groups were included, is likely to reflect the smaller inequalities in the oldest age groups but heterogeneity that was found in all health outcomes, particularly in the age group 85–94, is related to the healthier study sample in the DLSA study. Thus, it is likely that also summary estimates are somewhat underestimates of health inequalities in this study.

## Conclusions

This study showed the extent of inequalities in health and functioning among people aged 75+ in four Nordic countries. Despite the selection processes, health inequalities according to level of education persist in old age and, for mobility and ADL, also in the oldest age groups (95+). In the Nordic countries, known for their generous welfare systems, health inequalities have been observed in younger ages (Huijts et al. 2010), and this study suggests that they continue until the last years of life.
